# *Lactiplantibacillus plantarum* Ln4 Alleviates High-Fat Diet-Induced Obesity by Modulating Lipid Metabolism and Adipogenesis in C57BL/6 Mice

**DOI:** 10.3390/nu17233668

**Published:** 2025-11-24

**Authors:** Yunjung Lee, Na-Kyoung Lee, Nayoung Kim, Yong-Min Choi, Haebom Kim, Hyun-Dong Paik, Eunju Park

**Affiliations:** 1 Department of Food and Nutrition, Kyungnam University, Changwon 51767, Republic of Korea; hjlee@kyungnam.ac.kr (Y.L.); skduddl8238@naver.com (N.K.); 2 Department of Food Science and Biotechnology of Animal Resources, Konkuk University, Seoul 05029, Republic of Korea; nakyoung_lee@nate.com; 3 Daesang Wellife, Seoul 03130, Republic of Korea

**Keywords:** *Lactiplantibacillus plantarum*, high-fat diet, lipid metabolism, adipogenesis, obesity

## Abstract

Background/Objectives: This research examined the anti-obesity potential of *Lactiplantibacillus plantarum* Ln4 in C57BL/6 mice fed a high-fat diet (HFD) for eight weeks. Methods: Forty male mice were randomly assigned (*n* = 8 per group) a normal diet (ND), HFD, HFD with orlistat (positive control), or HFD supplemented with Ln4 at 5 × 10^8^ or 5 × 10^9^ CFU/day (Ln4-8, Ln4-9). Ln4 was orally administered once daily throughout the study period. Results: Ln4 supplementation significantly attenuated body weight gain, visceral fat accumulation, and dyslipidemia, while improving lipid metabolism and hormonal balance. The Ln4-9 group exhibited effects comparable to those of orlistat, indicating potent anti-obesity activity. Ln4 also reduced serum triglyceride and total cholesterol concentrations and improved lipid and hormone profiles. At the molecular level, Ln4 downregulated adipogenic and lipogenic regulators (PPARγ, SREBP1c, and C/EBPα) while upregulating genes involved in lipolysis and fatty acid oxidation (PPARα, CPT1, and ACO) in the liver and adipose tissue. These changes were accompanied by lower insulin and leptin levels and restored adiponectin concentrations. Conclusions: Collectively, the results demonstrate that *L. plantarum* Ln4 mitigates HFD-induced obesity by improving lipid metabolism, suppressing adipogenesis, and balancing metabolic hormones, suggesting its potential as a functional probiotic for obesity management.

## 1. Introduction

Obesity is a growing global health problem closely associated with metabolic disorders such as type 2 diabetes, cardiovascular disease, and dyslipidemia [1]. Excess energy intake, physical inactivity, and chronic low-grade inflammation contribute to abnormal lipid accumulation and insulin resistance [2]. Although pharmacological interventions are available, their long-term use is limited by gastrointestinal discomfort and nutrient malabsorption [3].

Accumulating evidence highlights the gut microbiota as a central regulator of host energy balance, lipid metabolism, and adipose tissue function [4,5]. Dysbiosis caused by high-fat diet (HFD) feeding has been linked to obesity and metabolic inflammation, whereas modulation of gut microbial composition through probiotic supplementation is considered a promising approach for metabolic control [6,7,8,9]. Recent systematic reviews indicate that the anti-obesity potential of probiotics is strain-dependent, and that effects depend on host–microbiota interactions and metabolic signaling pathways [10,11,12].

Among various probiotic species, Lactiplantibacillus plantarum is widely recognized for its antioxidative, anti-inflammatory, and lipid-modulating properties [13]. Notably, the *L. plantarum* Ln4 strain, isolated from Korean napa cabbage kimchi and registered as KCCM11897P, has demonstrated strong antioxidant capacity and inhibition of lipid accumulation in vitro [14,15]. However, the in vivo mechanisms underlying its anti-obesity effects remain unclear.

Recent studies have shown that other *L. plantarum* strains, such as LMT1-48 and Q180, exert anti-obesity effects by regulating lipogenic gene expression and suppressing adipocyte differentiation in both cell and animal models [16,17]. These findings highlight that *L. plantarum* can regulate energy balance through lipid metabolic and adipogenic pathways; however, whether the Ln4 strain exhibits similar or distinct metabolic effects in vivo remains to be elucidated.

Given the strain-specific variability and limited physiological data, this study aimed to evaluate the anti-obesity effects of *L. plantarum* Ln4 in an HFD-induced obesity mouse model and to elucidate its mechanistic pathways involving lipid metabolism, adipogenesis, and hormonal regulation. We hypothesized that Ln4 could mitigate diet-induced obesity by modulating lipid metabolic and adipogenic signaling through the gut–liver–adipose axis.

This in vivo validation will provide critical insights into the translational potential of Ln4 as a functional probiotic for obesity management.

## 2. Materials and Methods

### 2.1. Preparations of Bacterial Samples

*L. plantarum* Ln4 (Ln4) was isolated from napa cabbage kimchi in South Korea [14]. Ln4 was cultured in de Man–Rogosa–Sharpe (MRS) broth (Difco Laboratories, Detroit, MI, USA) at 37 °C for 18 h under anaerobic conditions (GasPak system, BD, Franklin Lakes, NJ, USA). The cultured cells were harvested by centrifugation at 5000× *g* for 10 min at 4 °C, washed twice with sterile phosphate-buffered saline (PBS; Hyclone, Logan, UT, USA), and resuspended in PBS [13,14,15]. The bacterial suspension was lyophilized and stored at −80 °C until use for animal experiments.

### 2.2. Mouse Models and Experimental Design

A total of 40 male C57BL/6 mice (5-week-old) were purchased from Koatech (Ansan-si, Republic of Korea). Mice were housed 3–4 per cage at 23 ± 2 °C and 53 ± 2% relative humidity with a 12-h light/dark cycle. Male mice were selected to minimize hormonal variations associated with the estrous cycle, which can influence metabolic outcomes. After one week of acclimatization, the mice were randomly assigned to each group using a computer-generated randomization schedule, resulting in five groups (n = 8):

(1) normal diet (ND, 10% kcal fat; Research Diets D12450 B); (2) high-fat diet (HFD, 60% kcal fat; Research Diets D12492); (3) HFD with orlistat (Orlistat); (4) HFD with 5 × 10^8^ CFU/day of Ln4 (Ln4-8; low-dose group); and (5) HFD with 5 × 10^9^ CFU/day of Ln4 (Ln4-9; high-dose group). Ln4 was administered once daily by oral gavage in 200 µL of sterile phosphate-buffered saline (PBS), while ND and HFD control groups received the same volume of PBS. The doses (5 × 10^8^ and 5 × 10^9^) were determined based on previous in vivo studies on *Lactiplantibacillus plantarum* showing dose-dependent metabolic benefits [17,18]. A high-fat diet was provided for eight weeks, a duration sufficient to induce stable obesity in diet-induced models [19,20]. Orlistat was administered at 30 mg/kg based on previously validated dose regimens demonstrating effective inhibition of lipid absorption in HFD-fed mouse models [21,22]. An HFD was administered for 8 weeks, during which orlistat (30 mg/kg) or Ln4 (5 × 10^8^ or 5 × 10^9^ CFU/day; administered at two different concentrations to evaluate dose-dependent effects) was co-administered. The sample size (n = 8 per group) was based on prior studies employing similar experimental designs and was sufficient to detect significant differences in metabolic parameters under HFD conditions. Animal welfare was monitored daily, and all efforts were to minimize suffering. At the end of the experimental period, all mice were fasted for 12 h. Adipose tissues were harvested for subsequent analyses. All animal procedures were conducted in compliance with the Institutional Animal Care and Use Committee (IACUC) guidelines of Kyungnam University (approval no. KUICA-24-07). Following the 8-week feeding period, mice were fasted for 12 h and anesthetized using isoflurane (4 mL/kg). Blood samples were obtained and centrifuged at 2000× *g* for 30 min to isolate serum. Liver and adipose tissues were collected for gene expression assays relevant to adipogenesis and lipid metabolism. Subsequently, organs were weighed and stored at −80 °C until further use.

### 2.3. Biochemical Analysis of Serum Samples

Harvested serum samples were analyzed for Triglycerides (TG), Total cholesterol (TC), high-density lipoprotein (HDL) cholesterol, and low-density lipoprotein (LDL) cholesterol. These factors were determined using a BioSystems determination kit (Barcelona, Spain), following the manufacturer’s instructions.

Serum insulin, adiponectin, and leptin levels were measured at 450 nm using a microplate reader (Epoch; BioTek Instruments Inc., Winooski, VT, USA) and an ELISA kit (BioVendor, Modrice, Czech Republic). Serum free fatty acid and glycerol were determined using commercial assay kit (Bioassay Systems, San Francisco, CA, USA) according to the manufacturer’s guidelines.

### 2.4. RNA Extraction and Quantitative Real-Time PCR

Total RNA was isolated from liver and epididymal adipose tissue using TRIzol reagent (Invitrogen, Waltham, MA, USA). cDNA was generated from 1 μg of RNA using M-MLV reverse transcriptase (Promega, Madison, WI, USA). Quantitative real-time PCR was subsequently conducted with 25 μL of SYBR Green master mix (PhileKorea, QuantiSpeed SYBR No-ROX kit, Seoul, Republic of Korea) on a CFX Duet real-time PCR system (BIO-RAD, Hercules, CA, USA). The PCR protocol consisted of an initial denaturation at 95 °C for 10 min, followed by 50 amplification cycles at 95 °C for 10 s, 55 °C for 20 s, and 72 °C for 20 s. Primer sequences for PPARγ, SREBP1c, C/EBPα, FABP4, ACC1, FAS, ACADs, ACO, PPARα, and CPT-1 are provided in Table 1. β-actin was used as a reference gene. The normalized target gene expression levels in the samples were calculated using 2^−∆∆CT^. Values were expressed as fold change compared to control and are represented as the mean ± standard error (SE) (n = 7).

### 2.5. Obesity-Related Protein Activity

The epididymal adipose tissue and liver tissues were homogenized with cold PBS (4×, pH 7.4) in ice bath. The tissue homogenates were centrifuged at 10,000× *g* for 5 min, and the resulting supernatants were collected for the quantification of PPARγ, SREBP1, C/EBPα, ACCα, ACADs, and AMPK levels. The PPARγ (Enzyme-linked Immunosorbent Assay Kit for Peroxisome Proliferator Activated Receptor Gamma, Cloud-Clone Corp, Katy, TX, USA), SREBP1 (Mouse Sterol Regulatory Element-Binding Protein-1 ELISA Kit, MyBiosource, Inc., San Diego, CA, USA), C/EBPα (Mouse C/EBP alpha ELISA kit, MyBiosource, Inc., San Diego, CA, USA), ACCα (Enzyme-linked Immunosorbent Assay Kit for acetyl Coenzyme A Carboxylase Alpha, Cloud-Clone Corp, Katy, TX, USA), ACADs (Mouse acyl-CoA Dehydrogenase ELISA kit, abbexa, Houston, TX, USA), and AMPKα1 (Enzyme-linked Immunosorbent Assay Kit for Protein Kinase, AMP Activated Alpha 1, Cloud-Clone Corp, Katy, TX, USA) activities were determined using commercial assay kit according to the manufacturer’s guidelines.

### 2.6. Statistical Analysis

All results are expressed as the mean ± SE, and this format was consistently applied to all measured parameters. To compare multiple groups, data were analyzed using one-way analysis of variance (ANOVA) followed by Duncan’s multiple range test using SPSS software 25 (IBM, Armonk, NY, USA). Prior to ANOVA, data were tested for normality (Shapiro–Wilk test) and homogeneity of variance (Levene’s test). Differences were considered statistically significant at *p* < 0.05. In all figures, different superscript letters (a, b, c, etc.) above the bars indicate significant differences among groups, as determined by Duncan’s test, while values sharing the same letter are not significantly different.

## 3. Results

### 3.1. Effect of Ln4 on Body Weight and Body Fat Weight in Mice

**Figure 1:**
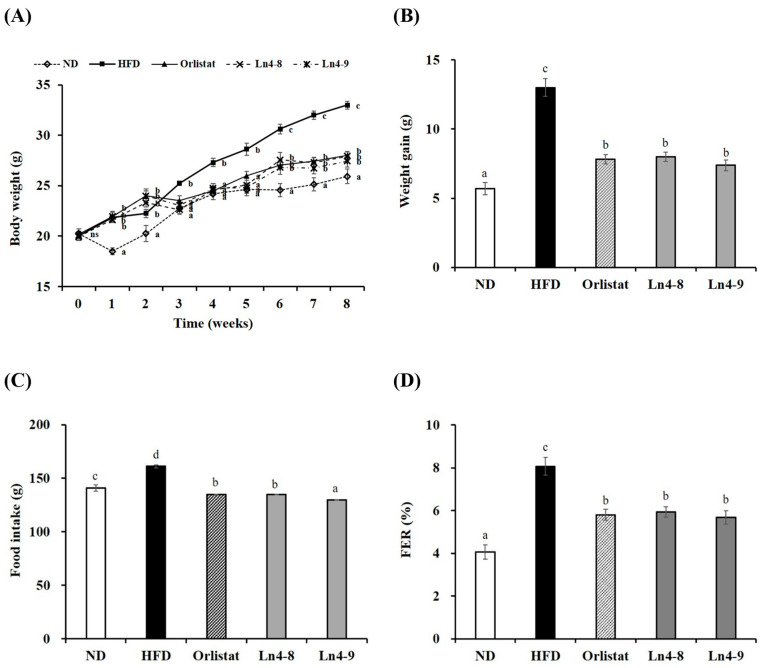
Effects of *Lactiplantibacillus plantarum* Ln4 on body weight change (**A**), weight gain (**B**), food intake (**C**), and FER (**D**) in mice. Food efficiency ratio (FER) was calculated by dividing body weight gain by food intake. Data are presented as mean ± S.E (n = 8). Statistical analyses were performed using one-way ANOVA followed by Duncan’s multiple range test, with significance set at *p* < 0.05. Distinct superscript letters (a, b, c, etc.) above the bars denote significant differences among groups. ND, normal diet (10% kcal fat); HFD, high-fat diet (60% kcal fat); orlistat, HFD with orlistat; Ln4-8, HFD with 5 × 10^8^ CFU/day of Ln4; Ln4-9, HFD with 5 × 10^9^ CFU/day of Ln4. Final body weight (**A**) and body-weight gain (**B**) were analyzed separately to distinguish cumulative weight change from overall weight increment during the 8-week period. Abbreviations: ND, normal diet; HFD, high-fat diet; Ln4-8, HFD + 5 × 10^8^ CFU/day Ln4; Ln4-9, HFD + 5 × 10^9^ CFU/day Ln4.

As shown in Figure 1, body weight increased progressively in all groups during the 8-week experimental period, with the greatest gain observed in the HFD group. In contrast, the orlistat-, Ln4-8-, and Ln4-9-treated groups showed significantly lower final body weight and body weight gain compared to the HFD group, indicating that Ln4 supplementation effectively attenuated HFD-induced weight gain.

HFD-fed mice also exhibited greater food intake and a higher food-efficiency ratio (FER) than ND-fed mice. Supplementation with Ln4 or orlistat significantly reduced both food intake and FER, suggesting improved metabolic efficiency under HFD conditions. Among these, the high-dose Ln4-9 group showed the lowest food intake, which was significantly lower than that of the HFD group, indicating that Ln4 supplementation may suppress excessive energy intake in a dose-dependent manner.

Relative adipose tissue weights are presented in Table 2, where visceral fat accumulation was markedly increased in the HFD group, while Ln4-9 treatment produced the most substantial reduction among the intervention groups.

### 3.2. Effects of Ln4 on Serum Lipids in Mice

High-fat diet feeding led to marked dyslipidemia, as shown by elevated serum TG, TC, and LDL-C levels compared with the ND group (Table 3). Ln4 supplementation significantly improved lipid abnormalities, with the Ln4-9 group showing the most pronounced reductions in total cholesterol and LDL-C levels. These findings indicate that Ln4 alleviates HFD-induced dyslipidemia and may modulate systemic lipid metabolism.

### 3.3. Effects of Ln4 on Adipocyte Differentiation and Lipid Synthesis in Livers and Epididymal Fat in Mice

**Figure 2:**
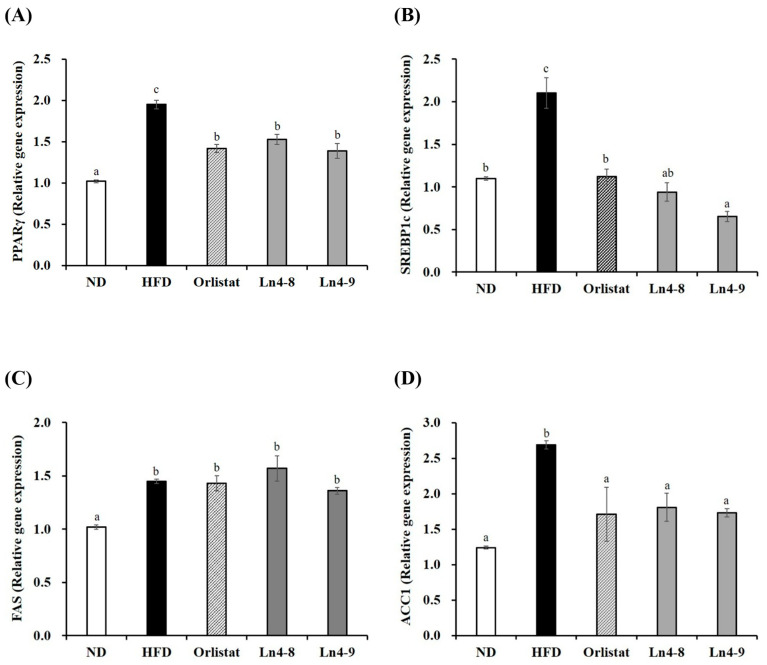
Effects of *Lactiplantibacillus plantarum* Ln4 on gene expression of adipocyte differentiation and fat synthesis in liver tissues. (**A**) PPARγ, (**B**) SREBP1c, (**C**) FAS, and (**D**) ACC1. Data are presented as mean ± S.E. of triplicate experiments. Statistical analyses were performed using one-way ANOVA followed by Duncan’s multiple range test, with significance set at *p* < 0.05. Different superscript letters (a, b, c, etc.) indicate statistically significant differences among groups. ND, normal diet (10% kcal fat); HFD, high-fat diet (60% kcal fat); orlistat, HFD with orlistat; Ln4-8, HFD with 5 × 10^8^ CFU/day of Ln4; Ln4-9, HFD with 5 × 10^9^ CFU/day of Ln4. Abbreviations: ND, normal diet; HFD, high-fat diet; Ln4-8, HFD + 5 × 10^8^ CFU/day Ln4; Ln4-9, HFD + 5 × 10^9^ CFU/day Ln4; PPARγ, peroxisome proliferator-activated receptor gamma; SREBP1c, sterol regulatory element-binding protein-1c; FAS, fatty acid synthase; ACC1, acetyl CoA carboxylase 1.

**Figure 3:**
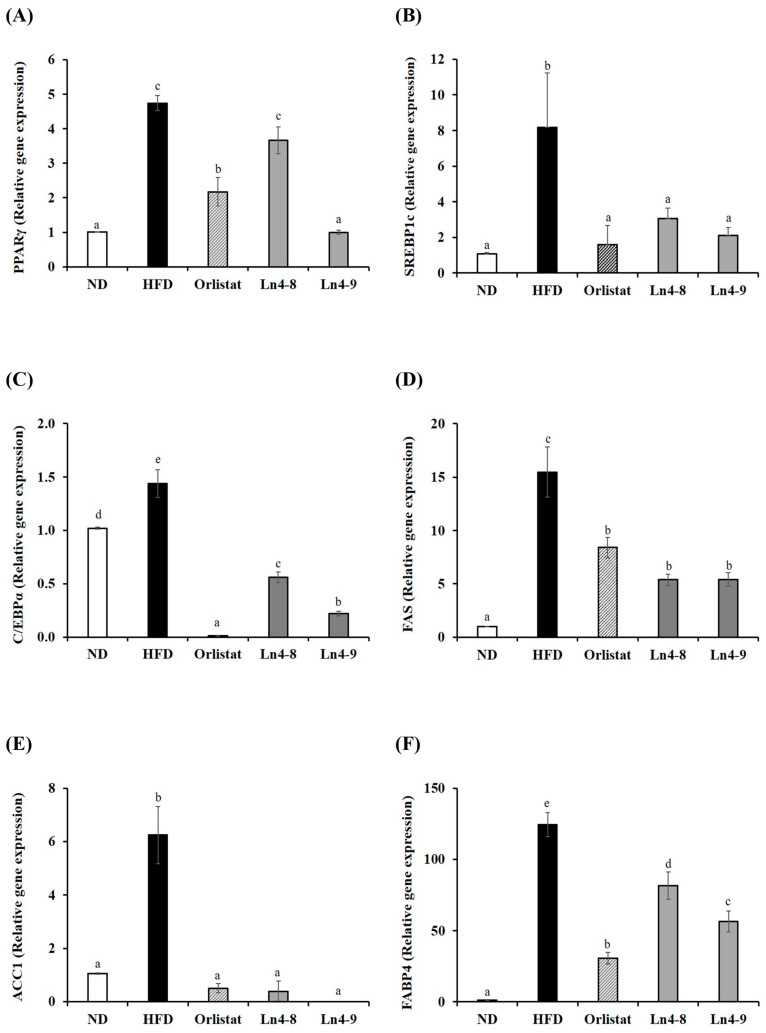
Effects of *Lactiplantibacillus plantarum* Ln4 on gene expression of adipocyte differentiation and fat synthesis in epididymal adipose tissues. (**A**) PPARγ, (**B**) SREBP1c, (**C**) C/EBPα, (**D**) FAS, (**E**) ACC1, and (**F**) FABP4. Data are presented as mean ± S.E. of triplicate experiments. Statistical analyses were performed using one-way ANOVA followed by Duncan’s multiple range test, with significance set at *p* < 0.05. Different superscript letters (a, b, c, etc.) indicate statistically significant differences among groups. ND, normal diet (10% kcal fat); HFD, high-fat diet (60% kcal fat); orlistat, HFD with orlistat; Ln4-8, HFD with 5 × 10^8^ CFU/day of Ln4; Ln4-9, HFD with 5 × 10^9^ CFU/day of Ln4. Abbreviations: ND, normal diet; HFD, high-fat diet; Ln4-8, HFD + 5 × 10^8^ CFU/day Ln4; Ln4-9, HFD + 5 × 10^9^ CFU/day Ln4; PPARγ, peroxisome proliferator-activated receptor gamma; SREBP1c, sterol regulatory element-binding protein-1c; C/EBPα, CCAAT/enhancer binding protein alpha; FAS, fatty acid synthase; ACC1, acetyl CoA carboxylase 1; FABP4, fatty acid-binding protein 4.

High-fat diet feeding markedly increased the expression of key transcription factors (PPARγ, C/EBPα, and SREBP1c) and lipogenic enzymes (FAS, ACC1, and FABP4) involved in adipocyte differentiation in both liver and epididymal fat tissues (Figure 2 and Figure 3). Ln4 supplementation significantly reversed these changes, particularly in the Ln4-9 group, which showed the most pronounced downregulation of adipogenic and lipogenic markers.

**Figure 4:**
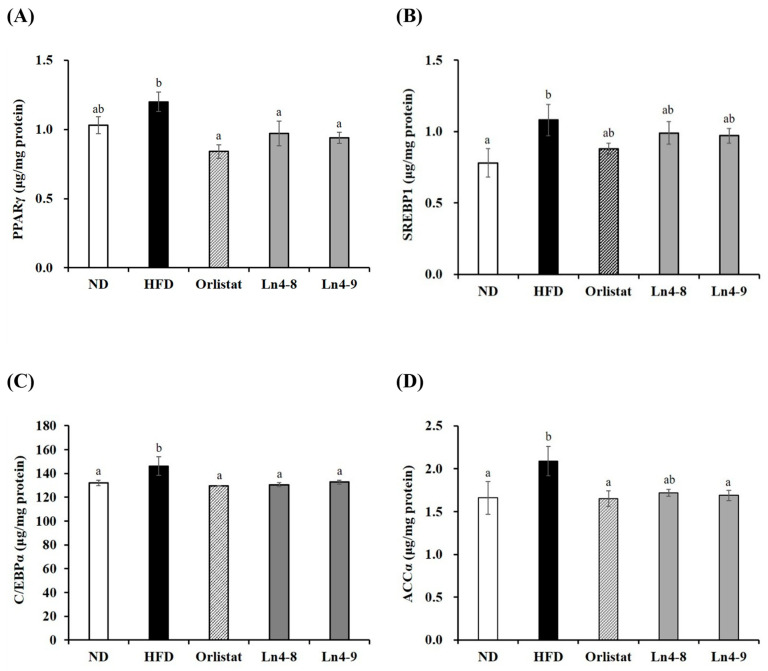
Effects of *Lactiplantibacillus plantarum* Ln4 on protein activity of adipocyte differentiation and fat synthesis in liver tissues. (**A**) PPARγ, (**B**) SREBP1c, (**C**) C/EBPα, and (**D**) ACCα. Data are presented as mean ± S.E. of triplicate experiments. Statistical analyses were performed using one-way ANOVA followed by Duncan’s multiple range test, with significance set at *p* < 0.05. Distinct superscript letters (a, b, etc.) above the bars denote significant differences among groups. Values that share at least one letter (e.g., “ab”) are not significantly different from groups labeled with either letter, whereas values with no letters in common differ significantly. ND, normal diet (10% kcal fat); HFD, high-fat diet (60% kcal fat); orlistat, HFD with orlistat; Ln4-8, HFD with 5 × 10^8^ CFU/day of Ln4; Ln4-9, HFD with 5 × 10^9^ CFU/day of Ln4. Abbreviations: ND, normal diet; HFD, high-fat diet; Ln4-8, HFD + 5 × 10^8^ CFU/day Ln4; Ln4-9, HFD + 5 × 10^9^ CFU/day Ln4; PPARγ, peroxisome proliferator-activated receptor gamma; SREBP1c, sterol regulatory element-binding protein-1c; C/EBPα, CCAAT/enhancer binding protein alpha; ACCα, acetyl CoA carboxylase alpha.

**Figure 5:**
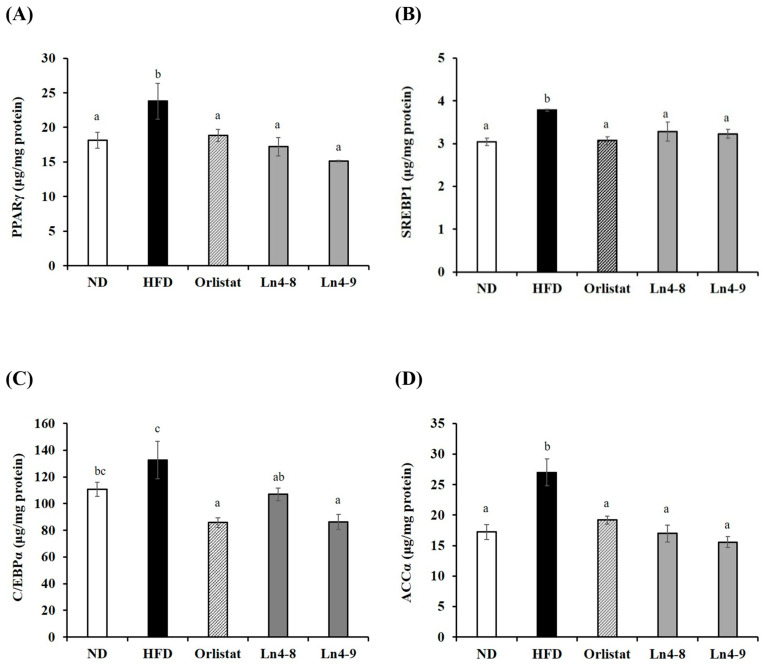
Effects of *Lactiplantibacillus plantarum* Ln4 on protein activity of adipocyte differentiation and fat synthesis in epididymal adipose tissues. (**A**) PPARγ, (**B**) SREBP1c, (**C**) C/EBPα, and (**D**) ACCα. Data are presented as mean ± S.E. of triplicate experiments. Statistical analyses were performed using one-way ANOVA followed by Duncan’s multiple range test, with significance set at *p* < 0.05. Distinct superscript letters (a, b, c, etc.) above the bars denote significant differences among groups. Values that share at least one letter (e.g., “ab”) are not significantly different from groups labeled with either letter, whereas values with no letters in common differ significantly. ND, normal diet (10% kcal fat); HFD, high-fat diet (60% kcal fat); orlistat, HFD with orlistat; Ln4-8, HFD with 5 × 10^8^ CFU/day of Ln4; Ln4-9, HFD with 5 × 10^9^ CFU/day of Ln4. Abbreviations: ND, normal diet; HFD, high-fat diet; Ln4-8, HFD + 5 × 10^8^ CFU/day Ln4; Ln4-9, HFD + 5 × 10^9^ CFU/day Ln4; PPARγ, peroxisome proliferator-activated receptor gamma; SREBP1c, sterol regulatory element-binding protein-1c; C/EBPα, CCAAT/enhancer binding protein alpha; ACCα, acetyl CoA carboxylase alpha.

Consistent with the mRNA results, protein expression analysis revealed tissue-specific regulation of adipogenic and lipogenic proteins (Figure 4 and Figure 5). In the liver, HFD markedly increased the levels of SREBP1, C/EBPα, and ACCα compared with the ND group, and these elevations were significantly reduced by Ln4 supplementation. In epididymal fat, HFD feeding also upregulated PPARγ, SREBP1, and ACCα, all of which were markedly downregulated following Ln4 treatment, with a dose-dependent decrease observed only for C/EBPα. These findings indicate that Ln4 attenuates lipid accumulation in hepatic and adipose tissues by inhibiting adipogenesis- and lipogenesis-associated pathways at both transcriptional and translational levels.

### 3.4. Effects of Ln4 on Lipolysis and Fatty Acid Oxidation in Livers and Epididymal Fat in Mice

**Figure 6:**
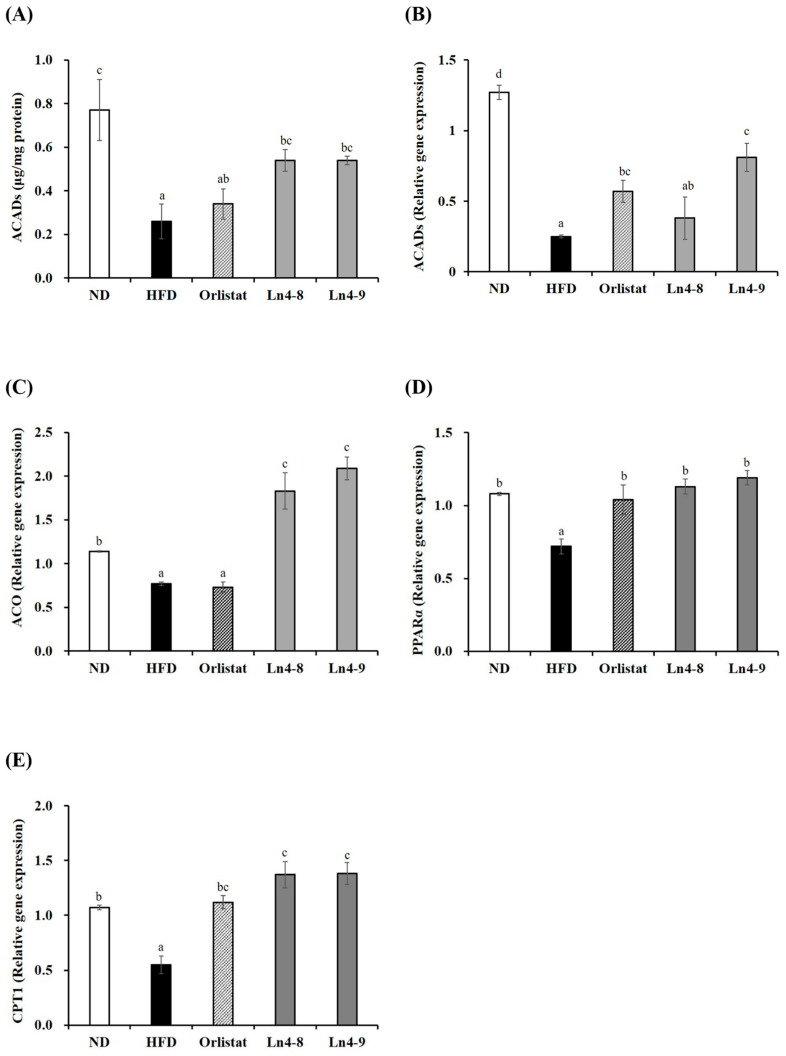
Effects of *Lactiplantibacillus plantarum* Ln4 on gene expression and protein activity of lipolysis and fatty oxidation in liver tissues. (**A**,**B**) ACADs, (**C**) ACO, (**D**) PPARα, and (**E**) CPT1. Data are presented as mean ± S.E. of triplicate experiments. Statistical analyses were performed using one-way ANOVA followed by Duncan’s multiple range test, with significance set at *p* < 0.05. Distinct superscript letters (a, b, c, etc.) above the bars denote significant differences among groups. Values that share at least one letter (e.g., “ab”) are not significantly different from groups labeled with either letter, whereas values with no letters in common differ significantly. ND, normal diet (10% kcal fat); HFD, high-fat diet (60% kcal fat); orlistat, HFD with orlistat; Ln4-8, HFD with 5 × 10^8^ CFU/day of Ln4; Ln4-9, HFD with 5 × 10^9^ CFU/day of Ln4. Abbreviations: ND, normal diet; HFD, high-fat diet; Ln4-8, HFD + 5 × 10^8^ CFU/day Ln4; Ln4-9, HFD + 5 × 10^9^ CFU/day Ln4; ACADs, acyl-CoA dehydrogenases; ACO, acyl-CoA oxidase; PPARα, peroxisome proliferator-activated receptor alpha; CPT1, carnitine palmitoyltransferase 1.

**Figure 7:**
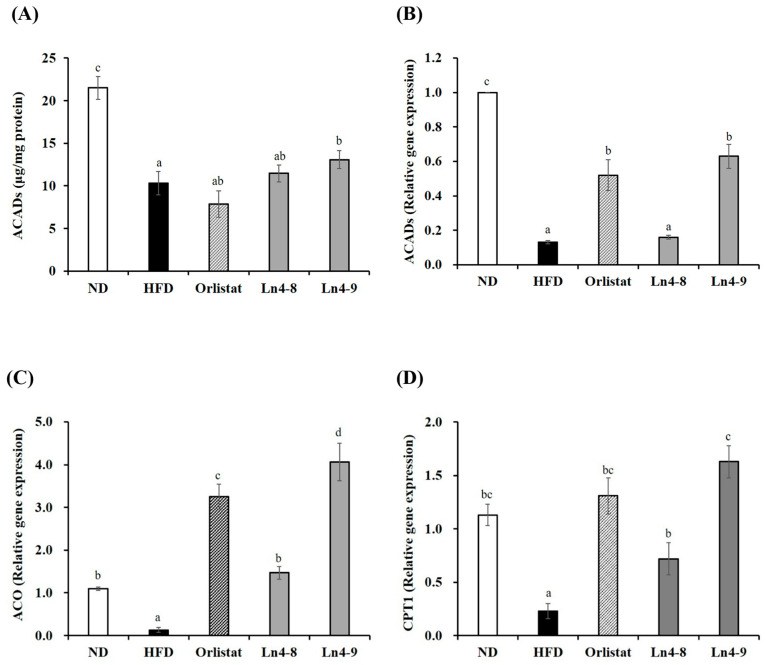
Effects of *Lactiplantibacillus plantarum* Ln4 on gene expression and protein activity of lipolysis and fatty oxidation in epididymal adipose tissues. (**A**,**B**) ACADs, (**C**) ACO, and (**D**) CPT1. Data are presented as mean ± S.E. of triplicate experiments. Statistical analyses were performed using one-way ANOVA followed by Duncan’s multiple range test, with significance set at *p* < 0.05. Distinct superscript letters (a, b, c, etc.) above the bars denote significant differences among groups. Values that share at least one letter (e.g., “ab”) are not significantly different from groups labeled with either letter, whereas values with no letters in common differ significantly. ND, normal diet (10% kcal fat); HFD, high-fat diet (60% kcal fat); orlistat, HFD with orlistat; Ln4-8, HFD with 5 × 10^8^ CFU/day of Ln4; Ln4-9, HFD with 5 × 10^9^ CFU/day of Ln4. Abbreviations: ND, normal diet; HFD, high-fat diet; Ln4-8, HFD + 5 × 10^8^ CFU/day Ln4; Ln4-9, HFD + 5 × 10^9^ CFU/day Ln4; ACADs, acyl-CoA dehydrogenases; ACO, acyl-CoA oxidase; CPT1, carnitine palmitoyltransferase 1.

High-fat diet feeding significantly reduced the expression of genes associated with mitochondrial fatty acid β-oxidation in both the liver and epididymal adipose tissue (Figure 6 and Figure 7). Ln4 supplementation effectively reversed these changes, particularly in the Ln4-9 group, which showed upregulated expression of key regulators including PPARα, CPT1, ACADs, and ACO compared with the HFD group. These effects were observed at both mRNA and protein levels, indicating enhanced fatty acid oxidation capacity following Ln4 treatment. In epididymal adipose tissue, Ln4-9 exhibited a more pronounced increase in ACADs expression, while both Ln4-8 and Ln4-9 consistently elevated CPT1 and ACO expression in a dose-dependent manner. Although increases in ACAD protein expression in adipose tissue did not reach statistical significance, the upward trend supports the transcriptional activation pattern. Taken together, these results suggest that Ln4 promotes fatty acid oxidation and reduces lipid accumulation in both hepatic and adipose tissues, contributing to its systemic anti-obesity effects.

### 3.5. Effects of Ln4 on Lipolytic Indicators in Livers and Epididymal Fat in Mice

**Figure 8:**
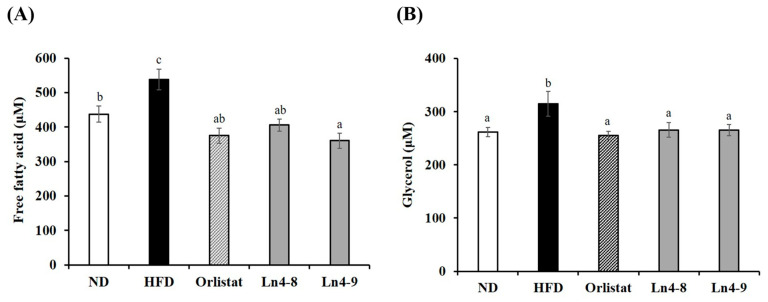
Effects of *Lactiplantibacillus plantarum* Ln4 on triglyceride degradation in serum. (**A**) FFA and (**B**) glycerol. Data are presented as mean ± S.E. Statistical analyses were performed using one-way ANOVA followed by Duncan’s multiple range test, with significance set at *p* < 0.05. Distinct superscript letters (a, b, c, etc.) above the bars denote significant differences among groups. Values that share at least one letter (e.g., “ab”) are not significantly different from groups labeled with either letter, whereas values with no letters in common differ significantly. ND, normal diet (10% kcal fat); HFD, high-fat diet (60% kcal fat); orlistat, HFD with orlistat; Ln4-8, HFD with 5 × 10^8^ CFU/day of Ln4; Ln4-9, HFD with 5 × 10^9^ CFU/day of Ln4. Abbreviations: ND, normal diet; HFD, high-fat diet; Ln4-8, HFD + 5 × 10^8^ CFU/day Ln4; Ln4-9, HFD + 5 × 10^9^ CFU/day Ln4.

High-fat diet feeding led to elevated circulating free fatty acids (FFAs) and glycerol levels, reflecting enhanced lipolysis under obesogenic conditions (Figure 8). Ln4 supplementation, particularly in the Ln4-9 group, significantly reduced FFA concentrations compared with the HFD group, suggesting attenuation of excessive lipid breakdown and improved metabolic regulation. In addition, glycerol levels were significantly lower in both the Ln4-8 and Ln4-9 groups than in the HFD group, although no significant difference was observed between the two Ln4-treated groups. These results indicate that Ln4 supplementation effectively mitigates HFD-induced elevations in circulating lipolytic markers, primarily through moderation of systemic lipid turnover. Collectively, Ln4 may help reduce lipid spillover and metabolic stress associated with obesity by lowering circulating FFA and glycerol levels.

### 3.6. Effects of Ln4 on Energy Metabolism in Mice

**Figure 9:**
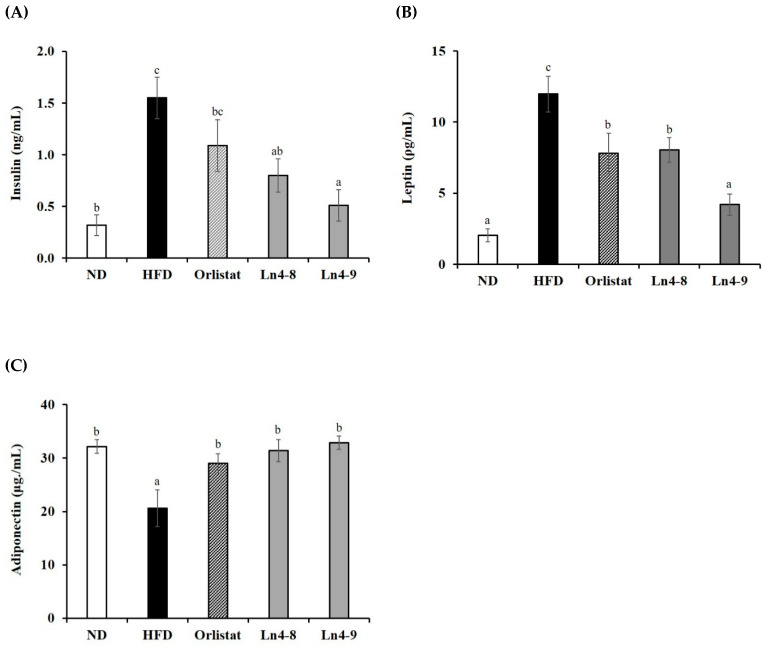
Effects of *Lactiplantibacillus plantarum* Ln4 on energy metabolism-related hormones in serum. (**A**) Insulin, (**B**) leptin, and (**C**) adiponectin. Data are presented as mean ± S.E. of triplicate experiments. Statistical analyses were performed using one-way ANOVA followed by Duncan’s multiple range test, with significance set at *p* < 0.05. Distinct superscript letters (a, b, c, etc.) above the bars denote significant differences among groups. Values that share at least one letter (e.g., “ab”) are not significantly different from groups labeled with either letter, whereas values with no letters in common differ significantly. ND, normal diet (10% kcal fat); HFD, high-fat diet (60% kcal fat); orlistat, HFD with orlistat; Ln4-8, HFD with 5 × 10^8^ CFU/day of Ln4; Ln4-9, HFD with 5 × 10^9^ CFU/day of Ln4. Abbreviations: ND, normal diet; HFD, high-fat diet; Ln4-8, HFD + 5 × 10^8^ CFU/day Ln4; Ln4-9, HFD + 5 × 10^9^ CFU/day Ln4.

**Figure 10:**
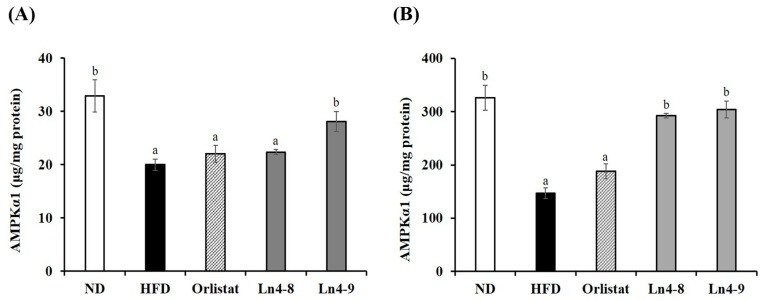
Effects of *Lactiplantibacillus plantarum* Ln4 on AMPK activity in (**A**) liver tissues and (**B**) epididymal adipose tissues. Data are presented as mean ± S.E. of triplicate experiments. Statistical analyses were performed using one-way ANOVA followed by Duncan’s multiple range test, with significance set at *p* < 0.05. Distinct superscript letters (a, b, etc.) above the bars denote significant differences among groups. ND, normal diet (10% kcal fat); HFD, high-fat diet (60% kcal fat); orlistat, HFD with orlistat; Ln4-8, HFD with 5 × 10^8^ CFU/day of Ln4; Ln4-9, HFD with 5 × 10^9^ CFU/day of Ln4. Abbreviations: ND, normal diet; HFD, high-fat diet; Ln4-8, HFD + 5 × 10^8^ CFU/day Ln4; Ln4-9, HFD + 5 × 10^9^ CFU/day Ln4; AMPKα1, AMP-activated protein kinase alpha 1.

High-fat diet feeding led to metabolic hormone dysregulation, as evidenced by increased serum insulin and leptin levels and reduced adiponectin concentrations compared with the ND group (Figure 9). Ln4 supplementation significantly restored these hormonal imbalances, with the Ln4-9 group showing the greatest improvement—insulin and leptin levels were markedly reduced, while adiponectin levels were restored to values comparable to the ND group. Further, AMPKα1 activity in both liver and epididymal adipose tissue was significantly reduced in HFD-fed mice, indicating impaired energy homeostasis (Figure 10). Ln4 supplementation reinstated AMPKα1 activity in a dose-dependent manner, with Ln4-9 showing the most pronounced effect. This activation of AMPK is consistent with the observed suppression of lipogenesis and enhancement of fatty acid oxidation, suggesting coordinated metabolic regulation. Taken together, these findings indicate that Ln4 improves systemic energy balance by restoring metabolic hormone profiles and activating AMPK-mediated signaling pathways, contributing to the overall anti-obesity effects observed in this study.

## 4. Discussion

Obesity is characterized by dysregulated lipid metabolism, adipocyte hypertrophy, and hormonal imbalance, contributing to metabolic diseases such as type 2 diabetes and cardiovascular disorders [23,24]. Probiotics have emerged as potential modulators of host metabolism through interactions with gut microbiota and lipid signaling pathways; however, their metabolic effects are known to be highly strain-specific and may vary in magnitude or direction depending on microbial genetics and metabolic capacity [25,26,27,28]. Therefore, evaluating strain-dependent functionality is essential when considering probiotic interventions for obesity management.

In this study, *Lactiplantibacillus plantarum* Ln4 supplementation for 8 weeks significantly reduced HFD-induced increases in body-weight gain and food-efficiency ratio (FER), particularly in the Ln4-9 group (Figure 1, Table 2). This reduction was accompanied by decreased visceral adipose accumulation, including epididymal, perirenal, and mesenteric fat depots. These findings align with previous reports that certain *L. plantarum* strains attenuate adiposity but also highlight dose-dependent effects, with Ln4-9 producing greater metabolic improvements than Ln4-8 [16,17].

Obesity is closely linked to dyslipidemia, characterized by elevated serum triglycerides (TG), total cholesterol (TC), and low-density lipoprotein cholesterol (LDL-C) [29]. Consistent with this, the HFD group exhibited elevated serum triglycerides and total cholesterol, whereas Ln4 supplementation improved serum lipid profiles, particularly in the Ln4-9 group (Table 3). These outcomes support prior evidence that probiotics can modulate lipid metabolism by altering bile acid–cholesterol cycling and hepatic lipid synthesis pathways [30,31].

At the molecular level, Ln4 suppressed adipogenesis and hepatic lipogenesis, as indicated by the downregulation of PPARγ, C/EBPα, and SREBP1c, along with reduced expression of their downstream enzymes FAS, ACC, and FABP4 (Figure 2, Figure 3, Figure 4 and Figure 5). These changes suggest attenuation of lipid biosynthesis at both transcriptional and translational levels, consistent with studies demonstrating anti-adipogenic effects of select *L. plantarum* strains [17,26,32].

Ln4-9 also enhanced fatty acid β-oxidation, as shown by increased expression of PPARα, ACADs, CPT1, and ACO in both liver and adipose tissues (Figure 6 and Figure 7). This coordinated upregulation suggests that Ln4 promotes mitochondrial fatty acid utilization rather than merely reducing lipid storage. Notably, similar effects have been reported for several *L. plantarum* strains, although strain-specific differences in metabolic activation have also been documented, indicating that the capacity to modulate β-oxidation is not universal among *Lactiplantibacillus* species [33]. In contrast, some probiotic strains show limited or inconsistent effects on lipid catabolism, emphasizing the importance of validating functional attributes at the strain level [34].

Moreover, enhanced β-oxidation is tightly linked to reductions in hepatic steatosis and adipocyte hypertrophy, suggesting a systemic shift toward improved lipid turnover and energy balance. These findings support that Ln4 modulates lipid metabolism through a coordinated regulation of lipogenesis and lipid catabolism, contributing to a multi-tissue improvement in metabolic flexibility and energy balance [16,35].

Serum free fatty acids (FFAs) and glycerol are commonly used indicators of systemic lipolysis, and their elevation under obese conditions is associated with increased fat mobilization, insulin resistance, and hepatic steatosis [36]. In this study, Ln4-9 significantly reduced both FFA and glycerol levels, indicating that Ln4 not only suppresses excessive lipolysis but also mitigates the metabolic stress associated with uncontrolled lipid mobilization (Figure 8). This coordinated reduction suggests that Ln4 may help stabilize adipose tissue lipid handling, thereby decreasing the release of lipid byproducts into circulation and preventing ectopic fat accumulation. Similar effects have been reported for select *Lactiplantibacillus plantarum* strains that promote adipose metabolic homeostasis and reduce peripheral lipolytic pressure [33]. However, such responses are not universal across strains, emphasizing the need for strain-specific validation of metabolic function [37].

Additionally, Ln4 restored adiponectin levels while reducing insulin and leptin, indicating improved systemic energy homeostasis (Figure 9). Because adiponectin activates AMPK, which inhibits SREBP1c-mediated lipogenesis and promotes fatty acid oxidation, increased AMPK activation observed in liver and adipose tissues (Figure 10) likely contributed to the observed metabolic improvements [38].

Although the present findings are robust, several limitations should be acknowledged.

First, only male mice were used, and sex-dependent metabolic differences should be investigated. Second, although probiotics are closely linked to gut microbiota remodeling and short-chain fatty acid (SCFA) production, gut microbial composition and metabolite profiles were not measured in this study [39]. Future studies integrating metagenomics and metabolomics will clarify whether Ln4-mediated metabolic improvements involve gut–liver–adipose axis signaling.

In summary, *Lactiplantibacillus plantarum* Ln4 exhibits strain-specific anti-obesity effects by: (1) suppressing adipogenesis and lipogenesis (PPARγ/SREBP1c axis), (2) enhancing fatty acid β-oxidation (PPARα/CPT1/ACO pathway), and (3) improving metabolic hormone balance and AMPK-mediated energy homeostasis.

These results support Ln4 as a promising functional probiotic candidate for obesity management, while emphasizing the need for future mechanistic and clinical validation.

## 5. Conclusions

In this study, *Lactiplantibacillus plantarum* Ln4 supplementation attenuated HFD-induced obesity in mice by reducing visceral adiposity, improving serum lipid profiles, and modulating key regulators of lipid metabolism. At the molecular level, Ln4 suppressed adipogenesis and lipogenesis (PPARγ, C/EBPα, SREBP1c, FAS, ACC, FABP4) while enhancing fatty acid β-oxidation (PPARα, CPT1, ACO) and restoring AMPK-mediated energy balance. These coordinated effects suggest that Ln4 exerts strain-specific metabolic benefits through the regulation of lipid synthesis, lipid catabolism, and hormonal homeostasis. However, this study was conducted only in male mice, and gut microbiota composition and SCFA profiles were not analyzed. Future studies incorporating microbiome and metabolomic analyses, as well as human trials, are warranted to clarify the translational potential of Ln4. Overall, Ln4 represents a promising probiotic candidate for obesity management, but its clinical applicability should be considered within the scope of preclinical evidence and further confirmed through mechanistic and human studies.

## Figures and Tables

**Table 1:** The primers related to obesity.

Targeted Gene	Sequence	
	Sense	Antisense
PPARγ	5′-ccacactatgaagacattccat-3′	5′-gttctactttgatcgcactttg-3′
SREBP1c,	5′-gtgtgcaccgtagttctggg-3′	5′-aggtcagcttgtttgcgatg-3′
C/EBPα	5′-cactatcgcctggaggac-3′	5′-cgttctgtgagcctgtga-3′
FABP4	5′-tttcccctagaaagcaatcc-3′	5′-agaaaatctgcacggtaagt-3′
ACC1	5′-ccctacacttactgatgagc-3′	5′-gggaagcaataagaacctga-3′
FAS	5′-aagaaagtgctggaaaagga-3′	5′-cagcaattctcgggatgtat-3′
ACADs	5′-gattcaaaatagccatgcaa-3′	5′-gcatacttcacagcacaatc-3′
ACO	5′-attaagtcgccaccattctt-3′	5′-ggtccgttgttactgaatct-3′
PPARα	5′-gaatccacgaagcctacc-3′	5′-gccatacacaaggtatcc-3′
CPT-1	5′-aagatcaatcggaccctaga-3′	5′-atagtcatgatgatcgaaac-3′

**Table 2:** Effects of *Lactiplantibacillus plantarum* Ln4 on body fat weight of HFD-fed mice. Values are expressed as mean ± SE (n = 8). Statistical differences among groups were determined by one-way ANOVA followed by Duncan’s multiple-range test (*p* < 0.05). “ns” indicates no significant difference among groups. Different superscript letters (a, b, c, etc.) indicate statistically significant differences among groups. Values that share at least one letter (e.g., “ab”) are not significantly different from groups labeled with either letter, whereas values with no letters in common differ significantly. ND, normal diet (10% kcal fat); HFD, high-fat diet (60% kcal fat); orlistat, HFD with orlistat; Ln4-8, HFD with 5 × 10^8^ CFU/day of Ln4; Ln4-9, HFD with 5 × 10^9^ CFU/day of Ln4.

Variable	ND	HFD	Orlistat	Ln4-8	Ln4-9
Relative Adipose Tissue Weight (g/100 g BW)					
Brown fat	0.31 ± 0.02 ^ns^	0.26 ± 0.01	0.29 ± 0.02	0.30 ± 0.02	0.26 ± 0.02
Subcutaneous fat	0.76 ± 0.09 ^a^	2.19 ± 0.18 ^b^	1.85 ± 0.18 ^b^	2.14 ± 0.13 ^b^	1.76 ± 0.16 ^b^
Visceral fat	1.68 ± 0.18 ^a^	6.94 ± 0.36 ^d^	5.27 ± 0.53 ^bc^	6.40 ± 0.50 ^cd^	4.69 ± 0.41 ^b^
Epididymal fat	0.99 ± 0.10 ^a^	3.83 ± 0.21 ^c^	3.06 ± 0.39 ^bc^	3.74 ± 0.33 ^c^	2.51 ± 0.29 ^b^
Retroperitoneal fat	0.23 ± 0.05 ^a^	1.29 ± 0.12 ^b^	0.99 ± 0.18 ^b^	1.20 ± 0.16 ^b^	1.05 ± 0.11 ^b^
Perirenal fat	0.12 ± 0.01 ^a^	0.47 ± 0.11 ^c^	0.28 ± 0.05 ^ab^	0.33 ± 0.04 ^bc^	0.21 ± 0.02 ^ab^
Mesenteric fat	0.35 ± 0.08 ^a^	1.35 ± 0.15 ^c^	0.94 ± 0.17 ^bc^	1.14 ± 0.10 ^bc^	0.91 ± 0.16 ^b^

Abbreviations: ND, normal diet; HFD, high-fat diet; Ln4-8, HFD + 5 × 10^8^ CFU/day Ln4; Ln4-9, HFD + 5 × 10^9^ CFU/day Ln4; BW, body weight.

**Table 3:** Effects of *Lactiplantibacillus plantarum* Ln4 on lipid profiles of HFD-fed mice. Values are expressed as mean ± SE (n = 8). Statistical analyses were performed using one-way ANOVA followed by Duncan’s multiple range test, with significance set at *p* < 0.05. “ns” indicates no significant difference among groups.Distinct superscript letters (a, b, c, etc.) above the bars denote significant differences among groups. Values that share at least one letter (e.g., “ab”) are not significantly different from groups labeled with either letter, whereas values with no letters in common differ significantly. ND, normal diet (10% kcal fat); HFD, high-fat diet (60% kcal fat); orlistat, HFD with orlistat; Ln4-8, HFD with 5 × 10^8^ CFU/day of Ln4; Ln4-9, HFD with 5 × 10^9^ CFU/day of Ln4.

Variable	ND	HFD	Orlistat	Ln4-8	Ln4-9
Triglyceride (mg/dL)	131.27 ± 0.51 ^a^	133.50 ± 0.86 ^b^	132.17 ± 0.44 ^ab^	131.64 ± 0.40 ^a^	131.71 ± 0.55 ^a^
Total cholesterol (mg/dL)	41.93 ± 0.50 ^ab^	45.61 ± 0.82 ^c^	43.57 ± 0.63 ^bc^	44.76 ± 0.81 ^bc^	40.31 ± 1.56 ^a^
HDL-cholesterol (mg/dL)	6.02 ± 0.45 ^ns^	5.98 ± 0.32	6.46 ± 0.53	7.13 ± 0.34	7.04 ± 0.40
LDL-cholesterol (mg/dL)	8.86 ± 0.76 ^a^	12.13 ± 0.34 ^c^	10.38 ± 0.42 ^b^	10.41 ± 0.33 ^b^	10.16 ± 0.46 ^ab^

Abbreviations: ND, normal diet; HFD, high-fat diet; Ln4-8, HFD + 5 × 10^8^ CFU/day Ln4; Ln4-9, HFD + 5 × 10^9^ CFU/day Ln4; HDL, high-density lipoprotein; LDL, low-density lipoprotein.

## Data Availability

The original contributions presented in this study are included in the article. Further inquiries can be directed to the corresponding author(s).

## Abbreviations

ACADs	Acyl-CoA dehydrogenases
ACCα	Acetyl CoA carboxylase alpha
AMPK	AMP-activated protein kinase
CPT-1	Carnitine palmitoyltransferase 1
FAS	Fatty acid synthase
PPARα	Peroxisome proliferator-activated receptor alpha
SREBP1c	Sterol regulatory element-binding protein-1c
ACC1	Acetyl CoA carboxylase 1
ACO	Acyl-CoA oxidase
C/EBPα	CCAAT/enhancer binding protein alpha
FABP4	Fatty acid-binding protein 4
HFD	High-fat diet
PPARγ	Peroxisome proliferator-activated receptor gamma
